# Publication outcomes of the abstracts presented at the 2011 European Congress on Osteoporosis, Osteoarthritis and Musculo-Skeletal Diseases (ECCEO-IOF11): A position paper of the European Society for Clinical and Economical Aspects of Osteoporosis, Osteoarthritis and Musculo-Skeletal Diseases (ESCEO) and the International Osteoporosis and Other Skeletal Diseases Foundation (IOF)

**DOI:** 10.1007/s11657-015-0216-5

**Published:** 2015-04-25

**Authors:** Véronique Rabenda, Olivier Bruyère, Cyrus Cooper, René Rizzoli, Fanny Buckinx, Adrien Quabron, Jean-Yves Reginster

**Affiliations:** Department of Public Health, Epidemiology and Health Economics, University of Liège, Liège, Belgium; MRC Lifecourse epidemiology Unit, University of Southampton, Southampton, UK; NIHR Musculoskeletal Biomedical Research Unit, University of Oxford, Oxford, UK; Division of Bone Diseases, Geneva University Hospital and Faculty of Medicine, Geneva, Switzerland; Department of Public Health Sciences and Bone Cartilage Metabolism Unit, University of Liège, Liège, Belgium

**Keywords:** Publication outcomes, ECCEO-IOF Meeting, Oral communication, Poster, Presentation format

## Abstract

**Summary:**

The publication outcomes of the abstracts presented during the ECCEO-IOF 2011 reflect a high research productivity, support the robustness of the selection process conducted by the Scientific Advisory Committee and suggest that IOF-ESCEO WCO is successful in its mission to promote and disseminate research.

**Background and Objective:**

The European (now World) Congress on Osteoporosis, Osteoarthritis and Musculo-Skeletal Diseases (IOF-ESCEO WCO, formerly ECCEO-IOF) is the largest worldwide event fully dedicated to the clinical, epidemiological, translational and economic aspects of bone, joint and muscle diseases. The role of the Scientific Advisory Committee is to select abstracts for oral communication or poster presentation based on a short summary of the research. The aim of the present survey was to determine the publication rate in international peer reviewed journals of abstracts accepted at the IOF-ESCEO WCO 2011 Meeting (formerly ECCEO-IOF11), the relationship, if any, between the presentation format of the abstract and its subsequent full publication and the impact factor of the journal in which research was published.

**Results:**

Of 619 abstracts accepted at the 2011 ECCEO-IOF11 annual meeting, 45 were accepted for oral communication and 574 accepted for poster presentation. In the subsequent 3 years (2011–2014), 191 abstracts were published as a full-length manuscript (30.9 %). The publication rate was significantly higher for oral communications (75.6 %) than for poster presentations (27.4 %; *p* < 0.0001). Publications derived from oral communications were published in journals with a higher impact factor (8.3 ± 10.1) than those arising from poster presentations (4.0 ± 2.3; *p* < 0.0001), but there was no difference in the time to publication (OC 16.3 [IQR 8.4–23.3] months vs PP 11.3 [IQR 5.3–21.4]; *p* = 0.14).

**Conclusion:**

These results indicate a high research productivity and an appropriate selection of oral communication by the Scientific Advisory Committee of ESCEO-IOF.

## Introduction

The European (now World) Congress on Osteoporosis, Osteoarthritis and Musculo-Skeletal Diseases-International Osteoporosis and Other Skeletal Diseases Foundation-European Society for Clinical and Economic Aspects of Osteoporosis, Osteoarthritis and Musculo-Skeletal Diseases (IOF-ESCEO WCO), formerly European Congress for Clinical and Economic Aspects of Osteoporosis-International Osteoporosis Foundation (ECCEO-IOF), is the largest worldwide event fully dedicated to the clinical, epidemiological, translational and economic aspects of bone, joint and muscle diseases. Researchers from various fields of health sciences are invited to submit scientific abstracts which are then assessed for relevance and scientific value by an independent Scientific Advisory Committee (SAC). Amongst the abstracts selected by the SAC, those obtaining the highest ranking are presented as oral communications (OC), while the others are given as poster presentations (PP) during dedicated sessions. Presentation of a study at international meetings allows for a more rapid distribution of current research information [1]. However, members of SAC select abstracts for OC or PP based only on a short summary of the research. The question arises whether the selection process for oral communications preferentially identifies high quality research. If so, then we hypothesized that the publication rates of subsequent full publications should be greater for oral presentations than for poster presentations [2]. In addition, the impact factor of the journals accepting full publications might be higher for oral presentations than for poster presentations. Thus, the aim of the present survey was to determine the publication rate of abstracts in international peer reviewed journals accepted at the IOF-ESCEO WCO 2011 Annual Meeting (formerly ECCEO-IOF11), the relationship, if any, between the presentation format of the abstract at the meeting and its subsequent full publication and the impact factor of the journal in which research was subsequently published.

## Material and methods

All abstracts (*n* = 619) that were accepted for presentation at the 2011 ECCEO-IOF Meeting were identified from the conference proceedings [3] and the meeting website. Abstracts were categorized by presentation format (i.e., OC or PP).

**Fig. 1:**
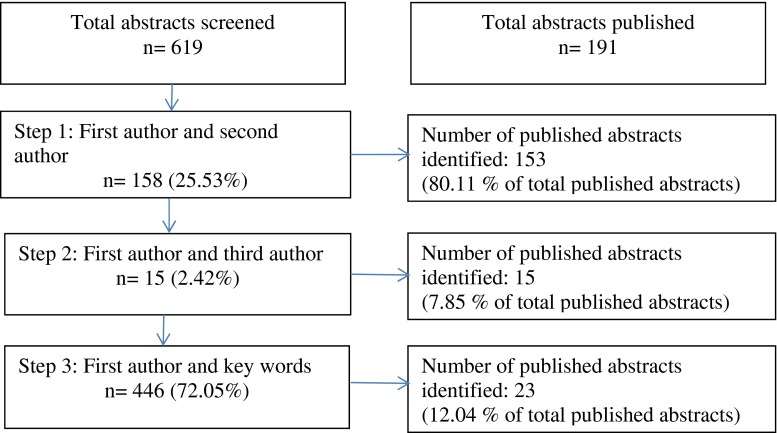
Research strategy results

Three investigators (FB, AQ and VR) conducted a manual search of the online PubMed database for each accepted abstract using a predefined search algorithm (Fig. 1). The start date was the abstract submission deadline. The first two steps of the algorithm searched published abstracts by entering the name of the first and second authors and the name of the first and third authors, respectively. If no published article was identified after steps 1 and 2, the search was reiterated with the first author’s name coupled to a list of keywords. These keywords were defined from the components of the abstract title. Their number ranged from a single keyword to as many keywords as needed to reflect the scope of the research. An abstract was considered published as a full paper if the title, authorship and, if available, the abstract of the full-length manuscript contain substantial similarities to the material appearing in the abstract published in the congress proceedings. If doubt persisted, the full-length manuscript was read in order to confirm adequate matching between the abstract presented at the Congress and the subsequent publication. Journal title, its impact factor (2012) and time to publication (months) were retrieved for each published article. The time elapsed between the presentation at the Congress and the publication of the manuscript was reported based on the “Epub ahead of print” and on the “Printed” publication dates. Descriptive statistics are reported as mean +/− SD or median and interquartiles range (IQR) for nonparametric variables.

For the comparison of the publication outcomes according to the presentation formats (OC vs PP), we used chi-square test for categorized variables and Student’s *t* test for continuous variables and Mann-Whitney *U* test for nonparametric variables. We calculated a weighted average impact factor in order to take into account the number of papers in of each journal. The journals which were more often cited were given more weight in the computation of the mean. In other words, the weight was allocated based on the number of times that the paper was cited.

## Results

From the 619 abstracts that were presented at the ECCEO-IOF11 Congress (45 OC and 574 PP), 191 (30.9 %) were published as a full-length manuscript. The three steps of the search strategy (Fig. 1) identified 153 (80.1 %), 15 (7.9 %) and 23 (12.1 %) of the full papers arising from the abstracts presented at the meeting. OC had a significantly higher publication rate than PP (75.6 vs 27.4 %; *p* < 0.0001), and the time to publication was significantly (*p* < 0.01) longer for OC when considering “Epub ahead of print” (*p* < 0.05; OC 14.8 [IQR 8.3–23.3] months vs PP 9.2 [IQR 2.7–17.7] months). However, the difference was no longer significant when considering the time to printed publication (OC 16.3 [IQR 8.4–23.3] months vs PP 11.3 [IQR 5.3–21.4]; *p* = 0.14). Approximately 15 % of the full papers were published between the deadline for submission of abstracts and the date of the Congress. Based on the “Printed” publication date, half of the papers were published within 12 months of their presentation, and almost 80 % of the publications were published within 2 years of the Congress; the remaining papers being published during the third and last year of the survey.

Abstracts presented at the ECCEO-IOF11 meeting resulted in full publication in 81 different journals. Table 1 shows the top 5 journals that were responsible for 46.6 % of all publications. The weighted average impact factor (IF) of the journals where manuscripts derived from the presentations occurring at the ECCEO-IOF11 congress were published was 4.8 + 5.1 (median 4.1; IQR 2.4–6.0) and ranged from 0.1 to 53.30. A highly significant difference in the journal impact factor (*p* < 0.0001) was observed between manuscripts associated with OC (8.3 ± 10.1) and PP (4.0 ± 2.3).

**Table 1:** Top 5 journals containing published abstracts from the ECCEO-IOF11 Meeting

Rank	Journal	Impact factor (2012)	Abstracts published (*n*)	Total published ECCEO-IOF 11 abstracts (%)
1	Osteoporosis International	4.580	33	17.28
2	Bone	4.023	21	11.00
3	Journal of Bone and Mineral Research	6.373	17	8.90
4	Calcified Tissue International	2.376	10	5.24
5	Journal of Clinical Endocrinology and Metabolism	5.967	8	4.19

## Discussion

The overall publication rate (30.9 %) of the abstracts accepted for presentation at the ECCEO-IOF11 is essentially similar to that previously reported for international conferences organized in various areas of health sciences [1, 2, 4–13], i.e., ranging from 11 to 78 % [2]. Our survey only covers 3 years following the meeting. Most of the publications dealing with similar outcomes report much longer follow-up duration, usually between 5 and 7 years [1, 5–9] and up to 9 years [11]. In the literature, the mean and median time from abstract presentation to publication are in the range of 18 months [1, 4, 6], and publications appearing more than 3 years after the meeting can contribute to up to 20 % of the overall publication rate [1]. Therefore, the figures reported here for the ECCEO-IOF11, whereas consistent with those found in the literature, are most likely to be underestimated.

OC presented at the ECCEO-IOF11 have a significantly higher publication rate than PP (75.6 vs 27.4 %). This difference is also reported for other conferences, within the same field of medicine [1] as well as in other fields [5, 6, 11]. OC also were associated with publications in journals with a higher impact factor than PP (8.3 vs 4.0), also in accordance with previous observations [1, 5].

Both the rate of publication of OC and the IF of the journals were full manuscripts derived from OC presented at ECCEO-IOF11 were published (75.6 % and 8.3) are amongst the highest cited in the literature and compare favorably even with the publication outcomes of the clinical oral presentations made at the American College of Rheumatology/Association of Rheumatology Health Professionals 2006 Annual Scientific Meeting (ACR/ARHP), the most prominent and respected International Conference in the field of Musculo-Skeletal Disorders (71.2 % and 7.1) [1]. The role of the Congress SAC is to assess the scientific merit of submitted abstracts, based on very limited information. These publication outcomes confirm the robustness of the selection process of OC made by the SAC prior to the ECCEO-IOF11 Conference.

Few differences are reported; in the time needed for publication between OC and PP, an observation already made for other meetings [1, 6]. The mean duration before publication of the abstracts was also within the expected range, with the understanding that, as previously mentioned, our survey was of shorter follow-up than those described in other publications [1, 6, 8, 10].

More than 23 % of the abstracts were published in *Osteoporosis International* and in *Calcified Tissue International*, two journals which are the official Journals of the *International Osteoporosis Foundation* (IOF), one of the scientific societies in charge of the program of the ECCEO-IOF11 Congress. Previous reports also highlight that abstracts presented at congresses organized by scientific societies are preferentially submitted for publication to the official journals of these particular societies [1, 4, 6, 7, 10].

Several authors have conducted surveys to identify the reasons given by investigators for not publishing their studies in peer reviewed journals [2, 14]. Lack of time, ongoing and incomplete studies and author or co-author problems were frequently reported as main reasons for non-publication. Unfortunately, this information was not available in our survey.

In conclusion, the publication outcomes of the abstracts presented during the ECCEO-IOF 2011 show an average publication rate of the overall abstracts of 30.9 % but a very high percentage of the oral communications (75.6 %) published within 3 years in journals with a high impact factor (8.3). These results reflect a high research productivity, support the robustness of the selection process conducted by the ECCEO-IOF11 Scientific Advisory Committee and suggest that the European (now World) Congress on Osteoporosis, Osteoarthritis and Musculo-Skeletal Diseases (IOF-ESCEO WCO formerly ECCEO-IOF) Congresses is successful in its mission to promote and disseminate research.
